# Perceptions on the feasibility of decentralizing phlebotomy services in community anti-retroviral therapy group model in Lusaka, Zambia

**DOI:** 10.1186/s12913-019-4386-5

**Published:** 2019-08-14

**Authors:** Mpanji Siwingwa, Selestine H. Nzala, Bornwell Sikateyo, Wilbroad Mutale

**Affiliations:** 1 Department of health policy and management, University of Zambia, School of public health, P.O BOX 50110 Lusaka, Zambia; 2 Department of medical education development, University of Zambia, School of Medicine, P.O BOX 50110 Lusaka, Zambia; 3 Department of bioethics, University of Zambia, School of medicine, P.O BOX 50110 Lusaka, Zambia

**Keywords:** Phlebotomy, CAG, HIV/AIDS, ART, Decentralization

## Abstract

**Background:**

The focus of the community anti-retroviral therapy Group model is on drug refill, adherence and support groups. However, laboratory services are completely neglected in this model, and stable patient still have to go to the clinic for blood draws after drugs refills from the community. Due to the introduction of new ART drugs, the guidelines now recommend the use of viral loads to guide decision in switching all patients from NNRTI to dolutegravir based first line ART regimens. But the national viral load testing coverage stands at 37% and and falls short of meeting the global UNAIDS and phlebotomy delivery system is congested. The purpose of this study was to identify the perceptions in decentralizing phlebotomy services into the community anti-retroviral therapy Group model.

**Method:**

A qualitative case study design was used. Data were collected through ten Focused group discussions among community anti-retroviral therapy Group members, community and health care workers at anti-retroviral therapy clinics and in-depth interviews with five key informants. Data were managed with the help of Nvivo version 10 and analyzed using thematic method.

**Results:**

Positive perceptions were identified as those which contributed to decongesting phlebotomy rooms, reduced missing phlebotomy appointments, work Load, and lost results. Improved quality of phlebotomy service delivery and testing coverage, innovative access to laboratory services and encouraged patient’s accountability. The negative perceptions were compromised sample integrity, inability to perform prevention control and patients less contact with clinicians.

**Conclusion:**

The study has demonstrated that decentralizing phlebotomy services within the CAG model has greater potential to improve the quality of services delivery for patients. In addition, it has perceived threats on the quality of specimen collected, patient’s safety, and health care.

**Electronic supplementary material:**

The online version of this article (10.1186/s12913-019-4386-5) contains supplementary material, which is available to authorized users.

## Background

According to WHO the number of people accessing antiretroviral therapy (ART) has increased rapidly since 2005 from 2.2 to an estimated 21.7 million people globally and Sub-Saharan Africa achieved the greatest increase in ART coverage by reaching 9 million people, to about 37% coverage [1–3]. Services for ART in Zambia have expanded rapidly in recent years such that at the end of 2018, it was estimated that more than 900,000 Human Immunodeficiency Virus (HIV) infected adults and children were currently receiving lifesaving Antiretroviral (ARVs) for HIV treatment [4]. The increase in ART access has triggered the renewed interest in community health workers and community health Assistants, as they may play an important role in scaling-up antiretroviral treatment for HIV/AIDS by taking over a number of tasks from the health care workers.

The Ministry of Health (MoH) of the Republic of Zambia (GRZ) is committed to achieving the 90–90-90 United Nations Programme on HIV/AIDS **(**UNAIDS) targets and is aware that the conventional human resources and physical infrastructure currently are not adequate to accommodate national scale up of ART. Hence, they have adapted the Differentiated service delivery (DSD) which is a client-centered approach that simplifies and adapts HIV services across the cascade in order to reflect the preference and expectations of various groups of people living with HIV (PLHIV) while also reducing unnecessary burdens on the health system. Community ART models are being implemented in Zambia to allow patients to administer the ARV from the community with only one person going to collect the drugs for others [4–6].

The Community-based ART Model is a delivery model for antiretroviral therapy in which ART care is delivered at a community-based site. CAG model emanated from Mozambique in 2012 and this was prompted by the country’s high attrition rates, limited number of ART clinics, an influx of patients and longer distances covered to a health facility. This model constituted a group of six people and every month a different group representative was chosen and travelled to the clinic to collect drug at ART clinic on behalf of the other group members. The drugs were administered in the community and each member only visited the clinic twice in a year unless if they had complications. Hence the focus was on drug refill, adherence and support groups [7].

The piloting of this model in Mozambique demonstrated that it had benefits on the patient, community and clinic. Hence it then spread to other countries such as Zambia, Zimbabwe, Malawi Uganda and South Africa and it’s been implemented in most countries within the sub-Saharan region. [8–11]. The model reduced the financial and time costs associated with frequent clinic visits, promoted community-based peer support, improved adherence to treatment [12, 13]. In addition, this package minimized clinic contact for clinically stable ART patients and redirected the limited resources towards managing unstable patients with complex clinical problems [14].

However, in Zambia, studies have shown that community health workers (CHW) programs face additional challenges. These challenges included high turnover, low motivation, inadequate supervision, insufficient compensation or incentives, and low recognition [15, 16]. These challenges may impede the integration of the community phlebotomy into the models if they are not holistically addressed.

Phlebotomy is the act or practice of drawing blood through venipuncture for the purpose of treatment and diagnosis and is a link between the laboratory and patient [17, 18]. Phlebotomists in Africa are mainly responsible for collecting and properly packaging specimens such as blood, sputum, urine, other body fluids, tissues, etc. In addition, they are also responsible for ensuring that acceptance criteria for specimen is followed to the latter prior to testing and analysis and are often the only laboratory professionals who have direct contact with a patient during a clinical visit ([19–21]. In many countries in Europe, phlebotomy is performed by doctors, nurses, laboratory staff and other healthcare professionals [22–24]. Phlebotomy has been noted to have potential risks and has expose health workers and patients to bloodborne pathogens, such as human immunodeficiency virus (HIV), hepatitis B virus (HBV), hepatitis C virus (HCV), and viral haemorrhagic fevers and dengue [25]. Blood samples collected poorly or wrongly usually yields inaccurate results and misleads the clinicians, and in most cases, patients are either misdiagnosed or inconvenienced by repeat the testing [26–29].

In Zambia collection of blood is done by the Medical Officers, Lay Counsellors, Nurses, Clinicians and Laboratory staffs and its used for testing and donation. This also shows that different cadres are capable of collecting bloods from either the community or bedside and guarantee a quality sample provided the cadre of interest is properly trained and certified. For instance, MoH Zambia has mandated the community health workers to collection specimens for EID tests and prepare the samples and store it for collection. [29, 30].

Tenofovir Alafenamide (TAF) has been recommended in the Zambia Consolidated Treatment Guidelines for Prevention and Treatment of HIV as the alternative to Tenofovir Disoproxil Fumarate (TDF) since it has improved kidney and bone safety but similar efficacy when compared to TDF [31]. The guidelines now recommend the combination of Tenofovir disoproxil fumarate, Lamivudine and Dolutegravir which is commonly referred to TLD whereas the Tenofovir alafenamide, Emtricitabine and Dolutegravir is referred to as TafED for patients on first line regimen. The guidelines recommend the use of viral loads to guide decision in switching from NNRTI to DTG-based first line ART regimens and now requires that all patients on NNRTI be switched to DTG based regimen. But the national viral load testing coverage stands at 37% and and falls short of meeting the global UNAIDS and MoH 90–90-90 goals deadline by 2020. This implies that about majority of patients (67%) on NNRTI based regimen do not have the viral load result and these patients cannot be switched to stronger and better drugs unless this test is done. In addition, more consented efforts are needed to improve the viral load testing coverage, and enable MoH switch patients to newer and better drugs [32, 33].

However, the implementation of differentiated service delivery models in Zambia is decentralized half way (focus on drug refill), phlebotomy services are completely neglected in the model of delivery system [7]. Hence, opportunity costs for blood draws are too high. On average, patients must travel long distances to the clinic with high transport costs to have blood draws and may lose an entire day of work productivity (arising from long waiting hours at the clinic). In addition, burnt out health care workers, poor relationships with health workers and clinic congestion can make a clinic environment unpalatable for patients [9, 10].

The aim of this study was to determine stakeholder’s perceptions on the feasibility of incorporating the collection of blood samples for routine testing into the CAG model in Lusaka, Zambia. This was with the view to improving testing coverage in resource limited areas. The study proposed that the community health workers collect patients’ blood samples during the community monthly meeting and then sends samples for testing to the laboratory. Then only patients whose blood show virological failure, ARV drug toxicity, and intolerance or unresolved and prolonged side effects need to be referred to the facility [9–11].

## Methods

### Study site

A qualitative study was conducted in Lusaka province at both rural and urban ART clinics and it has an area of 21,896 km^2^. Lusaka has a population of 2,191,225 and density of 100 persons per km^2^ as of 2010. The urban and rural ART facilities were 10 km and 100 km respectively away from the central business district (CBD). The sites provided a conducive environment for identifying the perceptions in decentralizing specimen blood collection from the community in the CAG model.

### Study design

A qualitative methodological case study approach was used to identify the perceptions in decentralizing the blood sample collection in the CAG model.

### Data collection

A qualitative case study using semi-structured questionnaire was carried out between January 2018 and May 2018 [34, 35]. The semi-structured questionnaire was developed for this study (Additional file 1). Ten (10) focus group discussions (FGD) and five (5) in-depth interviews (IDI) were conducted among the four main stakeholders involved in the CAG-model: (a) MoH policy makers (b) professional health care workers; (c) Community health workers; and (d) Patients on ART in groups (CAG members); (see Table 1). In addition, FGDs were conducted for health care workers (HCW), CHWs and community ART group (GAG members and IDIs for MoH policy makers (see Table 1 for more details).

**Table 1:** Participants of the focus group discussions and in-depth interviews

Stakeholder groups	Number of FGDs	Number of IDIs
	Province	
CAG members/patients	4	0
Community Health workers	4	0
Professional Health workers	2	0
Policy makers	0	5
TOTAL	10	5

The study recruited 18 years of age and above GAG member, who were not acutely ill, already utilizing CAGs model and on ART for at least three months. Then HCWs and CHWs who had worked in the rolling out of CAG model in the ART facilities were recruited. Patients who had inability to participate in the group activities due to cognition deficits or mental illness or inability to provide consent or unwilling to participate in study were excluded from this study.

### Data analysis

All audio-recorded IDI and FGD were simultaneously translated from local languages into English and transcribed verbatim using Nvivo 10 Qualitative research software (QRS) International, Doncaster, Vic., Australia). All transcripts were imported into Nvivo 10 for data organization. Analysis was conducted simultaneously with data collection, with initial analysis of early interviews informing the themes explored in those that followed. [36].

Thematic analysis was performed through the process of coding in six phases in order to create recognized, and meaningful patterns. These phases were familiarization with data, generating initial codes, searching for themes among codes, reviewing themes, defining and naming themes, and finally producing the final report [37].

Validity was attained by member checking, where the interviewees were given the interpretation and drafted report in order to check the authenticity, accuracy, credibility, and validity of the study. In addition, the Researcher also allowed the Research Assistants to read the transcript for participants to consent whether what had been written reflected what they had said, commented and gave some clarifications. Concerning reliability, the Researcher compared the findings with those of other Researchers [9, 10].

### Ethical considerations/approval

All study participants gave a written consent to participate in the study. Ethical approval to conduct the study was obtained from the University of Zambia Biomedical Research Ethics Committee (UNZABREC). Written permission was sought from MoH, at both Provincial and District Medical Office to carry out the study in the ART clinics and verbal permission was also obtained from the Heads of the ART clinics where data was collected.

## Results

### Characteristics of participants for focused group discussions and in-depth interviews

The Table 1 below shows the participants characteristics for FGDs and IDIs and numbers of participants recruited in this study. The study was conducted in Lusaka province at both rural and urban ART clinics.

Table 2 below shows the major themes and sub-themes that emerged from the data analysis and these themes were the basis for the presentation of the data.

**Table 2:** Positive perceptions on decentralizing phlebotomy services into CAG model

Major themes	Sub- themes/categories
Perceptions to decentralize phlebotomy	Positive perceptions to decentralize phlebotomy 1. Decongested phlebotomy rooms 2. Reduced work load 3. Innovative way of bringing lab services closer to the people 4. Improved quality of phlebotomy service delivery 5. Improve current model and patient life 6. Reduced lost results 7. Inspired patients to monitor each other’s blood draws 8. Reduced missing appointments for blood draws 9. Improved testing coverage and TAT 10. Increased productivity in the community

### Positive perceptions to decentralizing phlebotomy services into CAG model

The perceptions were identified and categorized as being either positive or negative perceptions. Table 2 summarizes the positive perceptions on feasibility of decentralizing the collection of blood samples in the CAG model.

1. Decongesting phlebotomy rooms

Despite patients using the current CAG model, most of the participants felt that it took a long period of time to have blood drawn from the patients. Patients still had to stand in long queues and wait for blood to be drawn. Most patients came as early at 05.00 h in the morning and had bloods drawn after and finished after 13.00 h, and they wasted the whole day waiting for blood to be drawn. Most of the time the phlebotomy room became so congested with patients waiting for blood draws and this made the work environment unpalatable for both patients and health workers. Most participants said that including phlebotomy into the CAG model would greatly improve in decongesting the phlebotomy room and reduce the traffic of patients at the clinic. This however they said would work well if blood is collected from the community and then referred to the laboratory for testing.

*“ … … is a good ideal, community phlebotomy will help to reduced congestion and work load at the clinic … … ” (* **CAG members, FGD, rural clinic, 01/02/2018).**

*“I think with the inclusion of lab services in CAG model it will definitely go a long way to reduce congestion at the health facility …* ***”*** **(CHW, FGD, Urban clinic, 24/01/2018).**

*“It’s a great model and it will greatly decongest the bleeding area and patients will spend less time at the clinic” (* **CAG members, FGD, urban clinic, 23/01/2018).**

2. **Reduced work load in the Phlebotomy room**

Inclusion of community phlebotomy into the community model was perceived as a good thing because it would help in reducing work load in the phlebotomy room. Since bloods would be collected from the community, most CAG member felt that the traffic of patients at the facility would reduce, then health workers would then have less work and stress and would focus more on patients who really needed their services. Here is what the CAG members had to say;

*“Yes, if lab services are included in CAG, will make a huge difference, because it will mean less people to be attended at the facility, less work load for the profession health workers … … ”* (**CAG members, FGD, rural clinic, 31/01/2018).**

*“The perception, impression is that it will reduce congestion and work load for the health professions” (* ***HCW, FGD, urban clinic, 23/01/2018).***

*“community phlebotomy if introduced into ART clinics will reduce staff work load and help clinicians focus or concentrate on sick or unstable patients”* ***(Policy-maker-3, IDI, MoH, 14/03/2018).***

3. **Innovative way of bringing laboratory services to the people**

Decentralizing Community phlebotomy into the current model was perceived as a brilliant ideal. This was so because most policy makers said that this would bring laboratory services closer to the people and it was in line with the current ART policies in Zambia. In addition, they said that the current Zambian HIV guidelines promoted and encouraged the decentralization of health services by using DSD models. Some of the participants especially the policy makers, perceived this as a creative way of bringing laboratory services as close to people as possible so long as all the bottles necks that came with it were addressed in the implementation process. Here is what they had to say;

*“Integrating community phlebotomy into the CAG model is an innovative way of bringing laboratory services closer to the people”* (**Policy-maker-3, IDI, MoH, 14/03/2018).**

*“The smart way of providing the same services to a population that is congested and resource constrained” (* **Policy-maker-4, IDI, MoH, 17/05/2018).**

4. **Improve the quality of phlebotomy service delivery**

Majority of CAG members said including community phlebotomy into the CAG would improve the quality of service delivery for phlebotomy services. Usually at the time the bloods are collected from the patients, the phlebotomy room is congested and health workers are stressed and overloaded with work and the quality of service they got was unpalatable. So, they said including phlebotomy into the CAG model, would be a great thing, because they expected better and quality phlebotomy services rendered (such as attendance and quality of services) to them by health workers. Below was what the CAG members had to say;

*“The inclusion of laboratory tests in this model will greatly improve the quality of service delivery in the community or the way health personnel attend to us in that this service will be happening once every six months for each group and the patient will have no excuse but to do the labs since they will be brought on their door step”* (**CAG member, FGD, Rural clinic, 01/02/2018).**

*“Yes, the inclusion of lab tests in the CAG model will improve the quality of service delivery in the community or the way health personnel attend to us” (* **CAG members, FGD, Urban clinic, 23/01/2018).**

*“If labs are included in the CAG model it will improve the quality of health service in the community. In fact, no one will be missing labs and everything will be available as it will be done in the community closer to people so you should quicken the commissioning, the sooner it is rolled out the better” (* **CAG members, FGD, Rural clinic, 31/01/2018).**

5. **Improvement of the current model**

Majority of the policy makers felt that inclusion of the phlebotomy into the CAG model would greatly improve the current model and the life of the patient. The standard of care demanded that each new patient had the viral load test done by six months and used it to determine whether a patient was stable or unstable. However, most of the patients did not have this test done and it was difficult for Clinicians to ascertain whether a patient was doing well or not without the viral load being done. The participants felt that this would ensure that viral loads were done and doctors/clinician used that to determine whether a patient was stable or not. In addition, they said that inclusion of phlebotomy services would enhance in the monitoring of the patient drug regimens for toxicities and efficacy.

*“Phlebotomy will probably make the most difference than anything else especially when you look at what our standard of care is, our standard of care is every patient should have a viral load by 6 months. It’s as close as you can get knowing how the patient is doing without lab services its next to impossible to implement DST model because that’s the basis of saying you are stable...”* (**Policy-maker-1, IDI, MoH, 24/03/2018).**

*“Inclusion of the phlebotomy services is a plus or good thing because it will improve and enhance the current community models” (* **Policy-maker-4, IDI, MoH, 17/05/2018).**

*“We as community workers support the idea, I think it is a great idea which if implemented will go a long way to improve the model and life of people”* **(CHW, FGD, Urban clinic, 23/01/2018).**

6. **Reduced loss of laboratory results**

The loss of results was a common problem in all the facilities visited during this study. According to the patients and health care workers, the bloods were collected and they somehow went missing/lost and the patient was requested to recollect the blood draws. Most participants felt that including blood draws into the CAG model would greatly help in reducing on the number of results lost or misplaced for the patient and would minimize the frequency of blood draw repeats.

*“Yes, I think that will be good because we will have hope and assurance that they will be finding our results and this has led to people taking a year without doing labs but if it is going to be done that way that they are followed in the community I think even the labs will be up to date”* **(CAG Member, FGD, urban clinic, 24/01/2018).**

*“Now with the CAG model we have introduced a system that all CAG patients need to be identified. Whenever blood is drawn by way of indication of the words “CAG” on the Lab request slip so that the samples are quickly worked on”* **(HCW, FGD, urban clinic, 24/01/2018).**

7. **Inspire patients to monitor each other’s blood draws and tests**

The other perception was on encouraging patients to take keen interest on what the laboratory tests entails and why they are important. Patients mentioned that their fellow patients would be very keen to know who was doing well using the laboratory tests and who wasn’t and also be encouraged to monitor each other’s blood draws. In the process they said would improve adherence to phlebotomy appointments and majority of patients would be consistence in drawing blood. When it came to viral load, patients liked to have a suppressed viral load (as sign that they were done well) and they encouraged fellow members to have such results. Here is what was said;

*“The way we can notice that our friend does not do the labs is by way of his or her status, you will notice that if someone is not improving the way he or she used to look has changed then you will be able to conclude that this person does not do labs to check there status or CD4/VL and the person’s appearance will change then you will be able to know that they are not doing their labs …* ***”*** **(CAG Member, FGD, urban clinic, 24/01/2018).**

*“It also makes it easy for the patients to understand some of the lab tests because in some instances patients who are not in CAG as you are going through their files you will notice that they have labs results and when you ask them whether they know about these results and why they tested they will not give accurate answer. They will say I was just told to do the labs. Now if these lab services are included in CAG model the CAG members do understand these labs and the model will be the best”* **(HCW, FGD, Rural clinic, 01/02/2018).**

*“This system has really helped suppress the high viral loads in the patients with high viral loads. This situation will encourage them, such that those that are virologically suppressed will be an inspiration to others”* **(HCW, FGD, Urban clinic, 24/01/2018)**

8. **Reduced missing blood draw appointments**

The majority of participants revealed that including phlebotomy into this model would reduce the missing of appointments for blood draws. That now patients did not have to shy away from going to phlebotomy because it was congested, but that patient’s blood would be collected in the community where it was less congested. They said this would in a big way enhance and encourage CAG members from missing appointments for blood draws.

*“Encourage the patients to remind each in the group not to miss blood draws and be consistent in drawing bloods and in the process will reduce missing blood draws”* (**HCW, FGD, Rural clinic, 02/02/2018).**

*“Labs should be included in the model so that we do the labs as a group to reduce on queuing up, loss of results and workload”* (**CAG member, FGD, Urban clinic, 23/01/2018).**

9. **Improved testing coverage and TAT**

The inclusion of phlebotomy in the CAG model would help in fighting suppression of the virus in patients. Most participants said that once phlebotomy was included the testing coverage and results turn-around time (TAT) would improve. In addition, most policy-makers and HCW also mentioned that this would improve testing coverage in both rural and urban facilities in Zambia. So, most policy-makers felt that to meet the 90–90-90 UNAIDS goals, especially for the last 90% for viral suppression what needed to be done was to include the phlebotomy services in the community to enhance testing coverage for HIV viral load to compliment the current tested mechanism.

*“It all depends on the viral load, even to analyze them because in the next five years if the viral load coverage remains at 33% it means 33 % of people will be eligible to go into some form of model. Nevertheless, if your coverage is at 33% how many of this 33% will be suppressed? I don’t have statistics. So, imagine 90% to mean only 90% of that 33% will be able to go into a model. So, it is dependent on viral loads and it is crucial”* **(Policy-maker-1, IDI, MoH, 24/03/2018).**

10. **Increased economic involvement**

The CAG members highlighted that the inclusion of phlebotomy services into the current model would definitely make patients spend less time at the bleeding room and the more they became productive and engaged in economic activities. GAG members said that for those who ran business it meant they had enough time to concentrate on their businesses and those who had formal employment at least they had to ample time to attend to their families and work-related matters. Here is what they had to say;

… . “*You don’t stay long on the queue, you leave the facility early you are assured of early exit then you have more time to go and do other productive things and also save finances if you come from a distant place” ...* (**CAG member, FGD, Rural clinic, 23/01/2018).**

… “*They are applicable to decongest the facilities and also to give our clients time to do other productive things”* (**Policy-maker-2, IDI, MoH, 20/02/2018).**

… . “*You don’t stay long on the queue, you leave the facility early you are assured of early exit then you have more time to go and do other productive things you also save finances if you come from a distant place” ...* (**CAG Member, FGD, Urban clinic, 24/01/2018).**

“*More time for the patients and they will be more productive there by providing for their families. Healthy communities and much economic development and prosperous communities”* (**CAG Member, FGD, Rural clinic, 31/01/2018).**

### Negative perception to decentralizing phlebotomy into CAG model

The negative perceptions were classified into various categories, namely, compromised sample integrity and patients limited contact with clinicians. Table 3 below summarizes the negative perceptions on feasibility of decentralizing the collection of blood samples in the CAG model.

1. **Compromised sample integrity**

**Table 3:** Negative perceptions on decentralizing phlebotomy services into CAG model

Major themes	Sub- themes/categories
Perceptions to decentralize Phlebotomy	Negative perceptions on decentralize phlebotomy • Lack capacity to monitor drug toxicity and efficacy • Incompleteness of the current model • Inability to perform prevention control

Some health workers and policy makers perceived the inclusion of phlebotomy into this model as compromised sample integrity. They said the obvious problems were that clerical errors would raise and samples collected for specific test came in wrong tubes. Some samples would be exposed to high temperatures and sunlight and this may negatively affect the result of the patient. In addition, they felt that some samples might come after the required stability time and the laboratory might reject those samples.

*“Integrity of quality of the specimens to some degree will be negatively affected since there is no one to monitor and ensure that the results are of good quality”* (**Policy-maker-4, IDI, MoH, 17/05/2018).**

2. **Patients less contact with Clinicians**

Some of the health care workers viewed the inclusion of phlebotomy services into GAG model as limiting contacts with the Clinicians and they were concerned that this took time for them to know when the patient was failing treatment and intervention was usually done late when the patient was ill and needed urgent attention. Here is what they had to say about it:

“*There is now less contact with the patients and it takes time to know when the patient started failing on treatment and it takes long time for clinicians to intervene”* **(HCW, FGD, rural clinic 01/02/2018).**

## Discussions

Majority of participants perceived decentralizing the phlebotomy services within the community model as beneficial for the patients, health workers, community and health systems. The general feeling was that this had perceived benefits and all the cadres agreed that it was a progressive idea and would help in health system strengthening. However, the disagreements were on the modalities, such us where (designated localities within the communities) such interventions can be done and how do we ensure the safety of the patient and quality collection of the specimens. Generally speaking, there was consensus amongst the cadres interviewed (patients, CHW, HCW and policy-makers) that the idea was pragmatic and it must be piloted in Zambia.

The findings on perception of decentralizing community phlebotomy within the CAG model were similar to what other studies did in on GAG model even though in those studies the focus was on drug refill and retention rates [7–10, 14, 37]. Decongesting the phlebotomy rooms (clinics) was the most talked about perception by almost all the participants; this was because it had perceived benefits at individual, clinic and community levels. At individual level the patients would spend less time during blood draws, less on transport money and in the process concentrate on other things such as working and improving on businesses. At the clinic level, the work load reduces such that now the clinicians would have enough time to focus on patients who were really sick (unstable). At the community level it helped in reducing stigma. These benefits however, were expected to continue happening when phlebotomy services are incorporated into the CAG model.

However, perceived threats were highlighted in this study. Some of the negative perception included the compromise on the quality of samples collected by community health workers [38] and this is similar to studies done in POC testing. Most laboratory personnel were concerned on maintaining the sample integrity and ensure that the blood drawn was of good quality and that it should reach the laboratory within the recommended time frame. The other issues that was of great concern was avoiding clerical errors and usage of correct tubes for the correct tests and this was similar to what others have found [26–28]. However, they mentioned that if the sample went beyond the stability time, had a lot of clerical errors and came into a wrong bottle, then that sample would be rejected and patient would be requested to submit a fresh sample. Hence there was need for the laboratory scientist to be proactive and disseminate standard operating procedures, laboratory hand book (summary of tests offered and requirements) and take a lead in training and sensitizing the phlebotomist. Also, refresher trainings must be mandated to phlebotomist and they must be accorded opportunities for exchange training programs.

Despite asserting that the decentralizing the phlebotomy services in the community models, there is need to also profusely consider the acceptability and feasibility of this intervention from the patients, health provider and community’s perspectives. However, it is known from literature that successful implementation and integration of intervention depends on the acceptability of service providers and beneficiaries of the that service [39, 40]. Some essential elements for interventions’ acceptability are content, context and quality of service and if these elements are met for beneficiaries (patients and community), then they are more likely to adhere to recommendations and to benefit from such interventions [41, 42]. In addition, from the health providers view (health professionals and researchers), the essential element for acceptability is on delivery of service. If an intervention has poor delivery services then that intervention may not be implemented as planned and consequently may have low acceptability [43, 44]. This study has demonstrated and highlighted the greater need to conduct acceptability and feasibility studies on integrating phlebotomy services into the CAG model.

Studies in Zambia have shown that the WHO health building block’s specific weaknesses have a cross cutting effect in the health system. For instance, addressing challenges in key areas such as health work force, drug supply, health financing, and information systems does not guarantee success but rather addressing the these challenge with a health systems approach which considers all the six blocks (service delivery, health workforce, health information systems, access to essential medicines, financing, and governance) in applying the solution is what guarantees success. Health systems thinking approach espouses the necessity to use wider approaches in assessing the performance of health system interventions. Hence, it would be prudent for Government through the MoH policy-makers, and its partners to utterly consider system thinking approach when piloting the integration of phlebotomy into the DSD models. Health systems thinking approach will mitigate the perceived barriers and challenges [45–47].

Community sensitization and health education campaigns (for both phlebotomist, health workers and communities) are key in the successful implementation and integration of the phlebotomy services with the community ART programs. A lot needs to be done to educate the community leaders, community and ensure that there is consensus and awareness of this program. The government through MoH needs to come with health promotion programs that would ensure that the community is adequately sensitized and reach levels where they also buy into the idea. Community-based studies have shown that lack of community sensitization, engagements and health education from the starting point leads to low outcomes of health implementation programs and in the process leads to low uptake of health care services [48–50].

Integrating Phlebotomy services into the CAG model has financial implications and implementers of such interventions such as MoH would seriously need to consider the following areas for financial support; supplies, human resources and transportation costs.

Phlebotomy would require that all the materials such as gloves, cooler boxes, collection bottles (red, green and purple bottles), vacutainer holders and needles are readily available at any given time. Such materials are essential to the success of this program. But the great news is that MoH under Medical supplies Limited, is stocking all these materials and distributes such stock across the country upon requests from the local facilities. Hence, they would be need to adjust stock and monthly consumption, as these supplies would be projected to rise.

The other part that has cost implication is that of deploying the community phlebotomist to their respective places. A motivated and incentivized work force is crucial for the successful implementation and smooth running of this intervention. To try and address some of these challenges faced in health service delivery, the Zambian Ministry of Health (MoH) developed the National Community Health Assistant Strategy (MoH, 2010). This strategy has formalized and standardize the role of CHWs in the health system and has creating a health cadre called Community Heath Assistants (CHAs). CHAs undergo a one year’s standardized training programme, employed by government and registered with a general nursing council regulatory body. They are mentored and work under the supervision of nurses in delivering health services on a task-shifting basis. According to this strategy of 2010, the Ministry plans to train about 5000 CHAs by 2020 and by 2016 they were 1403 CHAs working at 789 facilities in every rural district. The CHAs are thus better suited and capable of handling the community phlebotomy because they have more intense training, are recognized by MoH, are enumerated, better supervised and additional training will be easier. It is for the above reasons CHAs have been recommended for the use of for community phlebotomy.

The other piece is that community phlebotomist would be very mobile as they would facilitate in collection of blood within the community and this would require that they have transport costs met from clinic to the community and back. So again, the Government through MoH should consider the use of either motor bikes or bicycles for phlebotomists or other means for specimens for transportation. The mode of transportation must be cost-effective and time sensitive so that it should preserve the sample integrity and must therefore, be reliable.

Hence the community phlebotomist must know exactly what testing bottles are needed for what test and the volume required for each test. For instance, purple is only for CD4 count, FBC and HIV viral load, green is for biochemistry and red is for RPR and hepatitis. The integrity of the specimen is key and of outmost importance [31]. Failure to maintain the sample integrity would lead to wrong diagnosis and patient mismanagement. However, phlebotomy services would have to be regularly monitored and supervised by the laboratory staff for the purpose of quality assurance and quality control. This supervision would be needed and must be done very often to give confidence to the patients, health care workers and policy-makers.

Among some benefits mentioned in the study on inclusion of the phlebotomy services is that of improving the testing coverage for HIV viral load. Most of the participants especially the heath care workers and policy-makers feel that this would greatly improve in meeting the UN goals of 95% virologically suppressed patients. Most of the remote setting have little to no access to viral load and the inclusion of the lab services would imply that these services would be done elsewhere and improve patient treatment management. And if most patient are virologically suppressed the community would also benefit, in that the rate of transmission would also reduce and eventually reduce the rate of new infection [31].

Some of the limitations of this study were that it focused on only the CAG model. But the current HIV guideline [31] prescribes the usage of four community models to improve service delivery. The prescribed models include urban adherence group (UAG), out of facility managed individual, and in facility managed models and CAG. However, this study only focused on CAG and this was a weakness for this study. We hope future studies would look at all the four community models and provide further information to inform policy and add to the body of knowledge. In addition, all the four-model including CAG, only focus on the stable patients and neglect unstable patients and would like to see other studies explore the use of unstable patients in the community models.

## Conclusions

The study has demonstrated that decentralizing phlebotomy services within the CAG model have perceived benefits for the community, clinic and patients and has great potential to improve the quality of services delivery. It also showed that it had perceived threats on the patients and community health workers. However, there are still areas for further research, such as cost benefit analysis studies, identifying cadres capable of perform community phlebotomy and explore feasibility of integrating phlebotomy services in other DSD models.

## Recommendations

- Advocating for cost benefit analysis studies that would compare the proposed model versus the traditional model of performing phlebotomy from the facility.
- We strongly recommend that MoH should consider utilizing the CHAs when implementing the Zambian adopted DST models.

## Additional file

Additional file 1: Semi Structured questionnaire. Both the FGDs and IDs followed the questions as outlined in the semi structured questionnaire. (DOCX 13 kb)

## Data Availability

The datasets used and/or analyzed during the current study available from the corresponding author on reasonable request.

## Abbreviations

ACCA: Association of Chartered Certified Accountants
AED: Automated external Defibrillator
AIDS: Acquired Immune Deficiency Syndrome
ALT: Alanine Aminotransferase
ART: Anti-Retroviral Therapy
ARV: Antiretroviral
AST: Aspartate aminotransferase
CAG: Community Antiretroviral Therapy Group
cART: Combination Antiretroviral therapy
CBART: Community Based ART
CD4: T-lymphocyte bearing CD4 receptor
CDC: Centers for Disease Control and Prevention
CHAs: Community health Assistants
CHW: Community Health Workers
CP: Community phlebotomy
CREAT: Creatinine
DBS: Dried Blood Spot
DSD: Differentiated service delivery
DTG: Dolutegravir
EDTA: Ethylene Diamine Tetraacetic Acid
EID: Early Infant Diagnosis
FBC: Full blood count
GCLP: Good Clinical Laboratory Practice
GNCZ: General Nursing Council of Zambia
GRZ: Government of the Republic of Zambia
Hb: Haemoglobin
HBsAg: Hepatitis B virus surface antigen
HCW: Health Care Workers
HIV: Human Immunodeficiency Virus
HIVVL: HIV-1 Viral Load
HNP: HIV Nurse Practitioner
HPCZ: Health Professional Council of Zambia
HTC: HIV Testing and Counselling
MoH: Ministry of Health
MSF: Médecins Sans Frontières
NGO: Non-Governmental Organization
PI: Principal Investigator
PLWHA: People Living with HIV and AIDS
PM: Policy Makers
POC: Point of care
POCT: Point of care testing
PSI: Population service international
QA/QC: Quality Assurance / Quality Control
SSA: sub-Saharan Africa
TafED: Tenofovir alafenamide, Emtricitabine and Dolutegravir
TAT: Turnaround Time
TDF: Tenofovir Disoproxil Fumarate
UAG: Urban Adherence Group
UNDP: United Nations Development Program
UNESCO: United Nations Education, Scientific and Cultural Organization
UNFPA: United Nations Population Fund
UNICEF: United Nations Children’s Fund
UNZABREC: University of Zambia Biomedical Research Ethics Committee
WHO: World Health Organization
