# Supplementary material for: Perceptions on the feasibility of decentralizing phlebotomy services in community anti-retroviral therapy group model in Lusaka, Zambia
Source: BMC Health Serv Res. 2019 Aug 14;19:570. doi: 10.1186/s12913-019-4386-5 (PMC6694622; doi:10.1186/s12913-019-4386-5)
Supplement: Supplementary file 1 — Semi Structured questionnaire. Both the FGDs and IDs followed the questions as outlined in the semi structured questionnaire. (DOCX 13 kb) [file 12913_2019_4386_MOESM1_ESM.docx]

**SEMI STRUCTURED QUESTIONS**

# what is a CAG model and why is it important?

# How do you form the CAG groups?

# Do you think collection of blood for lab services would make any difference when they are included in the model and why?

# What is your perception, impression on integrating phlebotomy services into CAG model?

# What impact would phlebotomy integration have on the clinic, community, and individual levels?

# What would be the benefits of including HIV phlebotomy services in the current model?

# In your opinion who should be eligible to collect the blood in the community and why?

# Lastly but not the least, in your opinion do you expect phlebotomy services (labs) added in CAG in the near future?
